# Identifying key demographic parameters of a small island–associated population of Indo-Pacific bottlenose dolphins (Reunion, Indian Ocean)

**DOI:** 10.1371/journal.pone.0179780

**Published:** 2017-06-22

**Authors:** Violaine Dulau, Vanessa Estrade, Jacques Fayan

**Affiliations:** 1 GLOBICE-Reunion, Grand Bois, Saint Pierre, Reunion Island, France; 2 BNOI-ONCFS, Parc de la Providence, Saint-Denis, Reunion Island, France; Institute of Deep-sea Science and Engineering, Chinese Academy of Sciences, CHINA

## Abstract

Photo-identification surveys of Indo-Pacific bottlenose dolphins were conducted from 2009 to 2014 off Reunion Island (55°E33’/21°S07’), in the Indian Ocean. Robust Design models were applied to produce the most reliable estimate of population abundance and survival rate, while accounting for temporary emigration from the survey area (west coast). The sampling scheme consisted of a five-month (June–October) sampling period in each year of the study. The overall population size at Reunion was estimated to be 72 individuals (SE = 6.17, 95%CI = 61–85), based on a random temporary emigration (γ”) of 0.096 and a proportion of 0.70 (SE = 0.03) distinct individuals. The annual survival rate was 0.93 (±0.018 SE, 95%CI = 0.886–0.958) and was constant over time and between sexes. Models considering gender groups indicated different movement patterns between males and females. Males showed null or quasi-null temporary emigration (γ” = γ’ < 0.01), while females showed a random temporary emigration (γ”) of 0.10, suggesting that a small proportion of females was outside the survey area during each primary sampling period. Sex-specific temporary migration patterns were consistent with movement and residency patterns observed in other areas. The Robust Design approach provided an appropriate sampling scheme for deriving island-associated population parameters, while allowing to restrict survey effort both spatially (i.e. west coast only) and temporally (five months per year). Although abundance and survival were stable over the six years, the small population size of fewer than 100 individuals suggested that this population is highly vulnerable. Priority should be given to reducing any potential impact of human activity on the population and its habitat.

## Introduction

Achieving reliable estimation of wildlife demographic parameters is of prime importance in ecological research and in most programs involving management and conservation of animal populations. In highly mobile species, such as marine mammals, one of the main challenges is to produce estimates for populations that range over a spatial scale that is usually unknown and/or cannot be entirely surveyed. Small resident populations of dolphins are generally monitored using capture–recapture approaches, based on individual photo-identification [1]. This technique implies that a significant portion of the population can be individually identified (i.e. photographically captured) based on the presence of natural, perennial marks and scars on the dorsal fin. Capture histories of distinct individuals are then used to fit different types of population models. Closed-population models are known to produce the most reliable abundance estimates [2], but require population closure; the data must be collected over a period that is short enough to ensure there is no gain or loss of individuals in the population through mortality, birth or migration (i.e. immigration and emigration). Conversely, open-population models have largely been developed to produce estimates of demographic parameters that require long-term data, such as survival rates [2]. Both of these standard models (closed and open) rely on the assumption that the entire population range is being sampled (i.e. all individuals are available for capture in the survey area), which is rarely the case. In particular, when the survey area does not encompass the entire home range of the population, individual movements in and out of the survey area are expected to occur and might introduce bias.

To take advantage of both closed- and open-population models while accounting for animal movements in and out of the survey area, a sampling scheme known as Robust Design was developed to produce robust estimates of both survivorship and population size [3, 4, 5]. The Robust Design approach involves sampling the population of interest at two temporary scales and thus requires the data to be structured in “primary samples”, which are made up of several “secondary samples”. The primary samples are repeated over long intervals and represent open sampling sessions, used for survival estimates, while the secondary samples are closed sampling sessions, used for abundance estimates. The two-level sampling of the Robust Design method allows the temporary emigration of individuals from the survey area to be investigated, based on the concept that all members of the population (i.e. all individuals associated with the survey area) might not be present at each sampling session [5]. Thus, different to standard open and closed mark–recapture analyses, Robust Design models consider the individual capture probability as conditional upon presence in the survey area and estimate the probability that a member of the population is not exposed to sampling (i.e. temporary emigration). Robust Design models have recently been used to produce demographic parameters of small resident dolphin populations [6, 7, 8, 9, 10, 11, 12]. These studies further demonstrate the importance of accounting for temporary emigration when investigating the population dynamics of coastal, yet highly mobile marine mammals, as home ranges generally extend beyond local survey sites.

Because of their confined coastal habitat and high residency, Indo-Pacific bottlenose dolphins, *Tursiops aduncus*, are particularly well-suited for mark–recapture monitoring. Within its range, the species seems to exhibit strong year-round residency and occur in small, local populations of usually a few hundred individuals [8, 9, 11, 13, 14, 15, 16, 17, 18, 19, 20], although estimates of up to thousands of individuals have been produced in some regions [21, 22, 23]. The species is found almost exclusively near the shore, generally in waters less than 100 m deep [19, 24, 25, 26]. No offshore sightings have been documented, and little is known about any potential movement into the open ocean. Genetic analyses conducted in some regions have indicated that populations are largely isolated and highly structured, with fine-scale genetic differentiation observed over very short distances [27, 28, 29, 30, 31]. The confined coastal habitat, genetic isolation and small population sizes of the Indo-Pacific bottlenose dolphin make the species particularly vulnerable to human-induced impacts, and the local status of the species has raised concern in some areas of increased interactions with anthropogenic activities [32, 33, 34].

Off the oceanic island of Reunion, in the Indian Ocean (55°E33’/21°S07’), the local population of Indo-Pacific bottlenose dolphins is considered as under threat, mainly due to the ongoing and foreseen habitat degradation related to the economic development of this French overseas territory [35]. Being a young volcanic island, the underwater relief is very steep, confining the dolphins’ habitat to a very narrow band near the coastline, within 1.2 km from the coast on average [26]. The species is present year-round, with no apparent seasonal changes in sighting frequency [36]. Currently, a growing emphasis on coastal planning and an associated large number of development projects in the marine environment (e.g. harbour extension, embankments, road construction, and renewable energy) is leading to direct loss and/or fragmentation of the dolphins’ core habitat [26]. Likewise, being confined to coastal waters, the species appears to be particularly exposed to anthropogenic pollution discharged by rivers and surface runoff [37]. On the west coast of the island, the species is targeted daily by whale/dolphin-watching and swimming activity, which is currently unregulated in Reunion/France. More positively, a Conservation Management Plan for coastal dolphin species is being developed in Reunion, but reliable estimates of population abundance and survival are needed as part of this to establish appropriate conservation actions.

The first objective of the present study was to produce the most accurate estimates to date of abundance and survival for the Indo-Pacific bottlenose dolphin population of Reunion. This was done using photo-identification data collected over several years on the west coast of the island, and by setting appropriate sampling periods to comply with the Robust Design sampling scheme. The second objective was to investigate the occurrence and extent of temporary emigration within the population to provide insights into the movement patterns of the species around the island, which has implications for population size estimates [38]. Finally, a third objective was to assess any differences in temporary emigration between males and females, to further investigate sex-specific differences in movement patterns.

## Materials and methods

### Ethics statement

Data collection was carried out under a research license (ref#:A-974-03) granted by the French Ministry of Food, Agriculture and Fisheries. Skin biopsies were collected under a permit (ref#: MC/2009/336) provided by the French Ministry of Environment, and under the approval of the Cyclotron Reunion Ocean Indian Ethics Committee.

### Photo-identification

Dedicated photo-identification surveys of *T*. *aduncus* were conducted all year round from 2009 to 2014 in the coastal waters of Reunion Island. Surveys were conducted on 5–7 m motor boats launched from different ports on the island, at an average speed of 5–6 knots, in good sea-state condition (≤ 3 on the Beaufort scale) and with four to six observers onboard. Transects were not pre-defined but were directed to cover the bathymetry range of the habitat of *T*. *aduncus* (waters less than 100 m deep) [26, 36]. Upon an encounter, the GPS position was recorded, the group size and number of newborn calves were estimated and photographs were taken for individual identification. The aim was to photograph the dorsal fin of all individuals in the group, irrespective of obvious markings on the animals. Photographs were taken using a CANON 60D-90D reflex digital camera equipped with a 100–300 mm zoom lens. Individual identification was based on the shape and distinguishing marks (notches, nicks, scars) of the leading and trailing edge of the dorsal fins. Only good quality photographs, based on sharpness, contrast, angle and size, were used to reduce misidentification [9, 39]. Dorsal fins presenting no distinguishing features were classified as “unmarked” and not considered in the matching process. The best photographs of the distinctively marked dorsal fins were matched with the photo-identification catalogue of the species, maintained since 2004. A new “capture” was defined as the first identification of an individual, whose dorsal fin picture was included in the catalogue, and a “recapture” was defined as the re-sighting of an individual already identified in the catalogue. All captures/recaptures were systematically validated by an experienced observer (VD). In the catalogue, each dorsal fin was assigned a marking level, to report on individual distinctiveness. Dorsal fins were graded on a three-level scale: (1) poorly marked (very few and small features); (2) moderately marked (medium sized notches and nicks); (3) highly marked (large notches or amputation) [8, 11, 21, 40]. Only distinctly marked individuals with a marking level of (2) or (3) (hereafter referred as “distinct individuals”), allowing for reliable and consistent identification, were included in the capture–recapture analyses, to avoid misidentification errors and reduce the heterogeneity in individual capture probability.

### Individual sexing

Dedicated boat surveys were conducted during 2010–2013 to collect skin samples, using a Barnett-Panzer crossbow (150 lb) and specialised darts equipped with biopsy tips (5 × 15 mm) from Ceta-Dart. A photograph was taken at the same time as the biopsy, to allow for the identification of the sampled individual. DNA was extracted from the skin sample using Qiagen DNeasy kits and the gender of individuals was determine genetically by amplifying the ZFX/ZFY region of the sex chromosomes by polymerase chain reaction [41]. When molecular data were not available, sex determination was based upon a photograph of the genital area or upon supplementary observational data. Individuals were identified as males when an underwater photograph of the penis was obtained during mating behaviour. Females were identified based on repeated and consecutive sightings (photographically captured on three different days or more) with a dependent calf during the study period [9, 12, 20]. Other individuals were classified as “unknown sex”.

### Mark–recapture models

#### Robust Design

Pollock’s Robust Design method [3, 4, 5] was applied to estimate population parameters over multiple years (2009–2014). To do so, the available dataset was constrained to comply with the assumptions of the Robust Design approach, which are a combination of the assumptions used for closed- and open-population models [4]:

1. The study area does not vary between sampling sessions;
2. The population is closed to additions (birth, immigration) and deletions (death, emigration) across secondary sampling occasions;
3. Marks are not lost and are correctly recognized on recapture;
4. Individuals have no behavioural response to capture that could affect their subsequent probability of recapture (i.e. trap-dependence);
5. Survival probability is the same for all individuals;
6. All individuals of the population used the study area during the study period, but not necessarily during every primary sampling session (allowing for temporary emigration).

**Fig 1:**
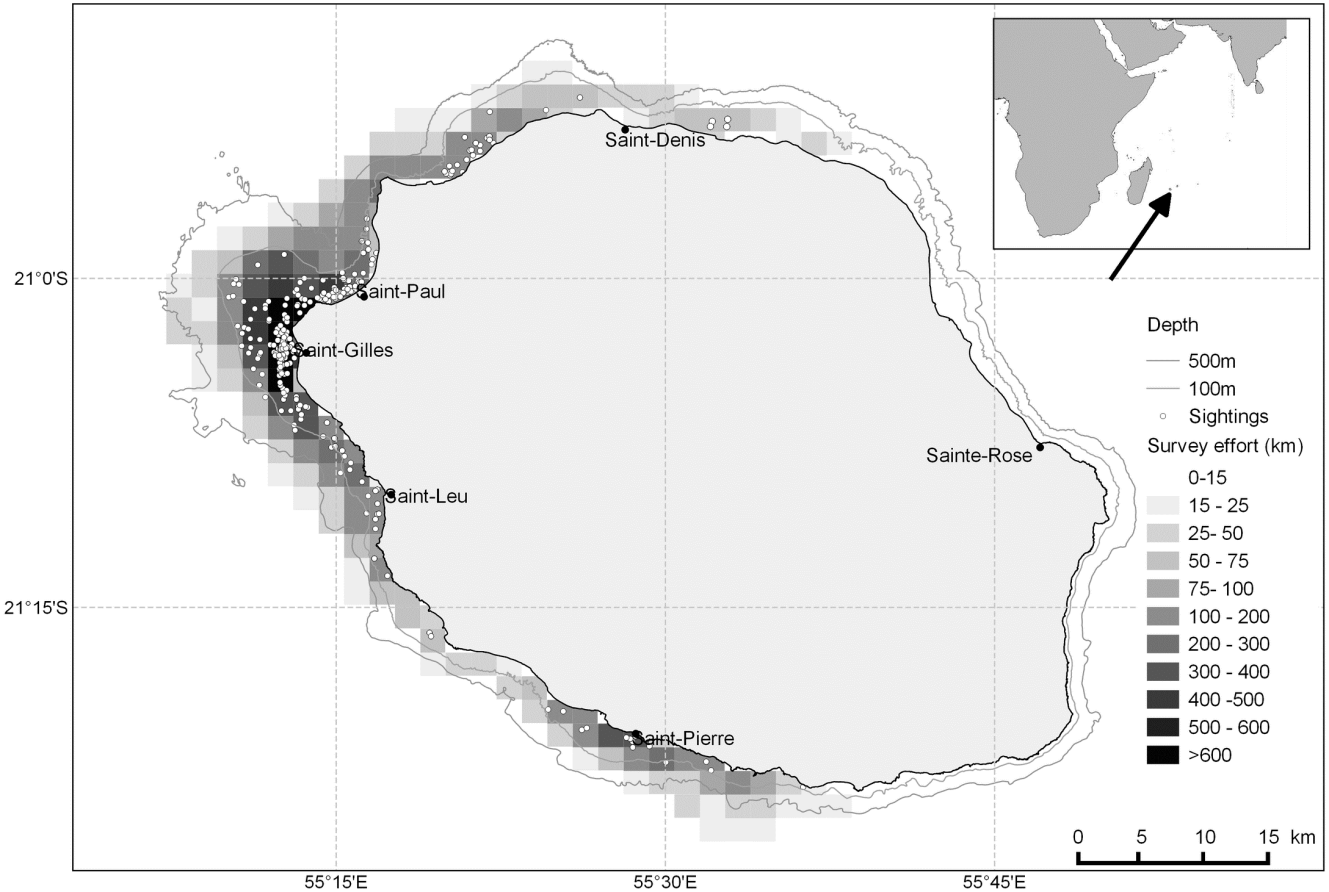
Map of Reunion Island showing the Indo-Pacific bottlenose dolphin sightings and the survey effort deployed on the west coast (study area) during June–October of 2009–2014.

Assumption (1) implies that the survey area is systematically covered on each sampling occasion. Therefore, although survey effort was deployed on the east coast in some years, the Robust Design study area was restricted to the western side of the island (Fig 1), which was consistently surveyed over the 2009–2014 period.

To fulfil the structure of the Robust Design methodology and achieve population closure across secondary sampling sessions [assumption (2)], the dataset was restricted to a five-month period in each year of the survey, during June to October of 2009–2014. This five-month period was selected after a series of closure tests to identify months where the assumption of demographic closure was met. Population closure tests were run in CloseTest, using the statistical test of Otis et al. [42], and using a significance level of 0.01. The Robust Design sampling scheme was thus defined *a posteriori*: the six years surveyed were treated as open primary sampling sessions (2009–2014), within which the five months sampled (June–October) represented the closed secondary sampling occasions.

Considering that only photographs rated from “good” to “excellent” quality were used, and only distinctly marked individuals [marking level of (2) or (3)] were included in the analysis, assumption (3)—regarding mark detectability—was believed to be reasonable. Although individuals were not physically captured and no attracting devices were used, the animals’ response to photographic capture (boat avoidance or attraction), which may have affected their subsequent probability of recapture, was tested [assumption (4)] using the trap-dependence test (TEST 2CT) in the U-care program [43]. No test exists to confirm the equal probability of survival among individuals [assumption (5)]. Regarding assumption (6), three around-island surveys were conducted in 2013 (S1 Appendix) to ensure that no other population was inhabiting the east coast, and thus that the overall population of Reunion was being sampled under the survey design.

The Robust Design models were implemented using the program Mark 6.2 [44]. The input data consisted of individual capture histories (i.e. binary matrix), constructed over 30 sampling occasions (five months per year over six years), during which individuals were recorded as present (1) if photographically captured at least once, or absent (0). The following parameters were estimated by the models:

- Annual apparent survival probability (*ф*);
- Probability of capture (*p*) and recapture (*c*) (as there was no indication of trap-dependence, *c* was set equal to *p*);
- Abundance of distinctively marked individuals (*N*_*d*_);
- Temporary emigration parameters, representing the probability that a member of the population is outside the survey area (not available for capture) during a primary session, given that it was previously present (*γ”*) or absent (*γ’*) from the survey area.

Models were developed based on three temporary emigration patterns [38]:

1. No temporary emigration: movement of individuals in and out the study area between primary sampling periods is null. Models were constructed by setting the two temporary emigration parameters equal to 0 (*γ” = γ’* = 0).
2. Random temporary emigration: animals move in and out of the study area randomly, so the probability of being outside the survey area is the same regardless of whether the animals were previously in (or out). Models were constructed by setting *γ”* equal to *γ’* (*γ” = γ’*).
3. Markovian temporary emigration: the probability of an individual to be outside the study area depends on previous states (in or out) [5; 45], so the two temporary emigration parameters were set as independent variables (*γ”*≠ *γ’*).

A series of models were constructed for each temporary emigration pattern, by allowing all parameters (survival, abundance and capture probability) to vary with time. More parsimonious models were then fitted to the data by constraining parameters to be constant. Standard model notations were used, with the subscripts (.) and (t) representing constant and time-dependent parameters, respectively. For capture probability, (t) and (T) referred to variation within and between primary sessions, respectively. The effect of survey effort on capture probability was also modelled, by incorporating the number of kilometers covered in waters less than 100 m deep and the number of survey trips conducted per secondary sampling occasion, as covariates. The influence of the distinctiveness of the dorsal fin was also tested, by using the marking level (2 or 3) as an individual covariate. These combinations resulted in a total of 116 models.

Models were compared and selected using the Akaike Information Criterion, adjusted for small sample size (AICc [46]). After correcting for the number of parameters, models showing the lowest AICc were identified as best fitting the data [47]. Models showing a difference in AICc of less than 2 (ΔAICc < 2) were also considered as providing support to the data and were thus also presented and discussed [47].

#### Robust Design with gender groups

To assess differences in survival and movement patterns between males and females, a Robust Design analysis was run using the same dataset but by pooling individuals into three distinct groups (female, male, unknown sex) in Mark 6.2. Models were developed, based on the three temporary emigration patterns, as described above, using a combination of parameters (survival, capture probability, temporary emigration) set to constant, time- or group- varying. The abundance parameter (*N*_*d*_) was set to vary between groups.

### Proportion of distinct individuals and overall population size

The capture–recapture models provided an estimate of the total number of distinct individuals (*N*_*d*_) within the survey area. The total number of individuals (*N*), including both distinct and unmarked individuals, was derived from the proportion of distinct individuals in the population (*θ*). As the group size of *T*. *aduncus* was relatively small (fewer than eight individuals on average; Table 1), *θ*_*i*_ was computed for each sighting *i*, by dividing the number of distinct individuals photo-identified [based on a mark level of (2) and (3)] by the total number of individuals [40, 48]. The mean (*θ*) was then calculated using only sightings made in optimal weather conditions (sea state of ≤ 2 on the Beaufort scale) and with a consistent team of observers, to ensure that all individuals in the groups were photographed and counted.

**Table 1:** Summary of the survey effort deployed over the study period (2009–2014), from June to October, off the west coast of Reunion, and the photo-identification effort applied to *T*. *aduncus*: Total and mean number of groups sighted with associated photo-identification data and mean group size.

	Number of survey days	Mean number of survey per month (± SE)	Survey effort (in km) in <100m deep waters	Number of groups sighted	Mean number of group per month (± SE)	Number of distinct individuals	Cumulative number of newly identified individuals	Mean group size (± SE)
2009	154	21.8 ± 5.1	2,479	35	7.0 ± 0.8	44	44	7.0 ±0.4
2010	199	28.2 ± 6.4	3,525	47	9.4 ± 2.0	42	52	5.7 ±0.3
2011	239	29.4 ± 3.8	3,523	78	15.6 ±2.3	42	56	6.3 ±0.3
2012	224	28.4 ±3.8	3,903	55	11.0 ± 1.5	48	62	7.4 ±0.3
2013	187	25.8 ± 6.2	3,215	45	9.0 ± 1	40	63	6.9 ±0.4
2014	170	23.8 ± 5.5	2,516	45	9.0 ± 2.7	44	66	6.8 ±0.4

The total number of individuals in the study area (*N*) was computed by dividing the number of distinct individuals (*N*_*d*_) estimated from the most parsimonious models, by the proportion of distinct individuals in the population (*θ*):

$$N=\frac{Nd}{\theta }$$

The standard error (SE) and 95% confidence interval (95%CI) of *N* were calculated using standard formulae [8, 12, 48], as detailed in S2 Appendix.

The abundance of the overall population of Reunion (*N*°) was extrapolated by taking into account the proportion of temporary emigrants. In the presence of random temporary migration (*γ”*≠ *γ’*), the abundance of the overall population associated with the survey area (*N°*) could be estimated as follows [5, 49]:

$$N{}^{\circ}\;=N\;\frac{1}{\left(1-\gamma ”\right)}$$

where *N* is the total number of individuals (distinct plus unmarked) present in the survey area (as computed above) and *γ”* is the probability of temporary emigration, estimated by the model. The SE for the overall population size (*N°*) was derived from the variance of *N* [50],

$$SE\left(N{}^{\circ}\right)=\sqrt{N^{2}\left(\frac{SE{\left(\gamma ”\right)}^{2}}{{\left(1-\gamma ”\right)}^{2}}+\frac{SE{\left(N\right)}^{2}}{N^{2}}\right)},$$

and the 95%CI was calculated assuming a log-normal approximation, with a lower limit of *N°*_*low*_ = *N°* / *C* and *N°*_*up*_ = *N°* × *C*, where [51]

$$C=\text{ exp}\left(1.96\;\sqrt{\text{ln}\left(1+\frac{SE{\left(N{}^{\circ}\right)}^{2}}{{\left(N{}^{\circ}\right)}^{2}}\right)}\right).$$

## Results

### Survey and photo-identification effort

**Fig 2:**
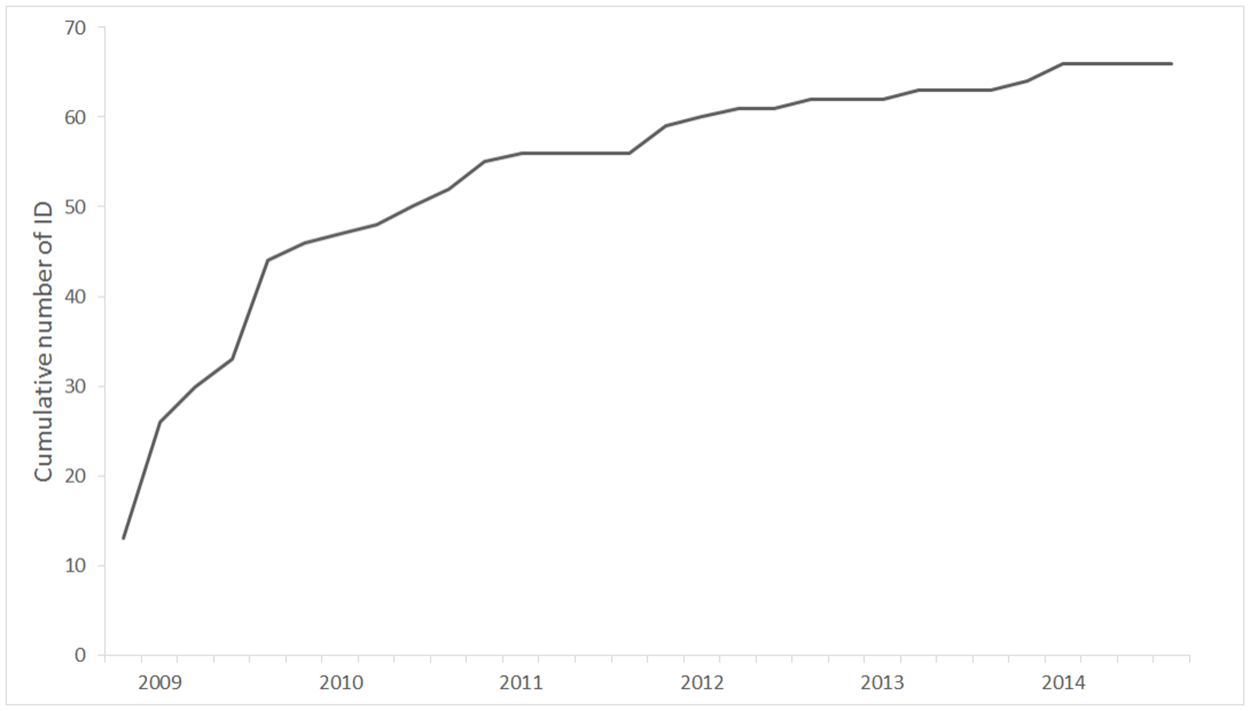
Cumulative number of newly identified individual dolphins [marking levels (2) and (3)], by secondary period (June–October), from 2009 to 2014, off the west coast of Reunion.

The sampling effort included a total of 1 173 daily surveys conducted off the west coast during the study period (June–October, 2009–2014), with an average of 26.2 surveys achieved per month from June to October (i.e. primary sampling period) (Table 1 and S1 Table). Between 2 479 and 3 903 km of survey effort was achieved in waters less than 100 m deep each year (Table 1 and S1 Table). A total of 305 sightings of *T*. *aduncus* with associated photo-identification data were completed (Fig 1). Between 35 and 78 groups were photographed during primary sampling sessions, representing a mean of 10 groups sighted per month from June to October each year. The mean group size was relatively small, ranging from 5.7 to 7.4 individuals (Table 1). The number of distinct individuals identified during primary sessions ranged from 42 to 48. The cumulative number of individuals identified during the study period was 66, with only four individuals newly identified within the last two years (Table 1, Fig 2). Of these 66 distinct individuals, 24% showed moderate markings [marking level (2)], and 76% were highly marked [marking level (3)]. The cumulative number of individuals identified during the sampling period levelled off, suggesting that the majority of distinct animals using the survey area were photographically captured during the course of the survey.

**Fig 3:**
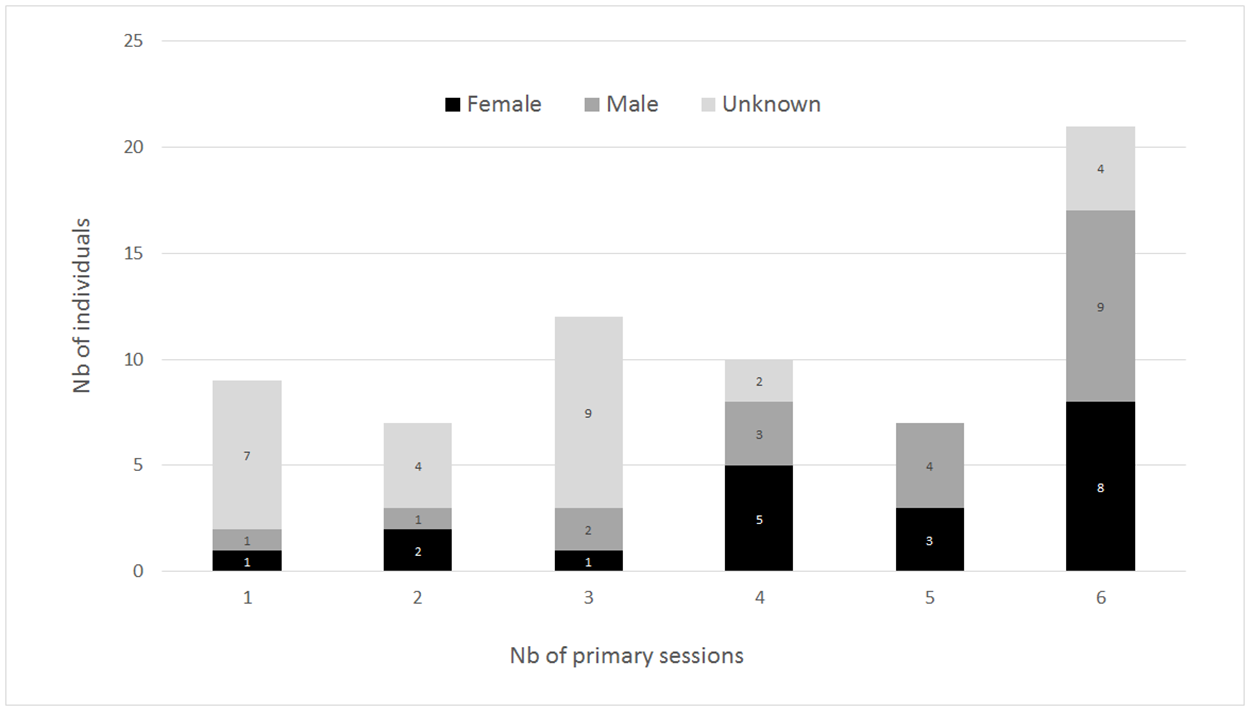
Distribution of the number of individual dolphins sighted in one to six primary sessions (2009–2014) off the west coast of Reunion, according to gender (female, male or unknown).

**Fig 4:**
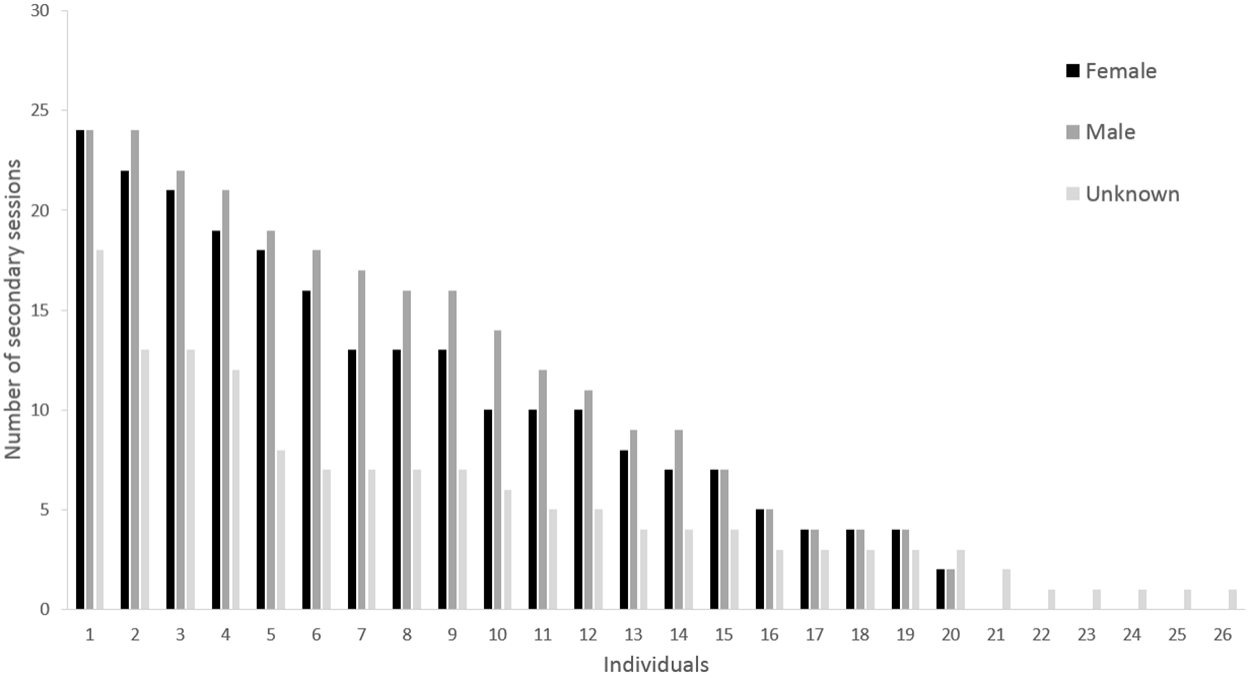
Distribution of the number of secondary sampling sessions (months) during which each male, female and unknown-sex individual was sighted (total number of secondary sampling sessions = 30).

Among the 66 distinct individuals, 20 were identified as females (11 from molecular sexing and nine from association with a young calf) and 20 as males (16 from molecular sexing and four from pictures of an erect penis), while gender was classified as “unknown” for 26 individuals. Most individuals (76%, 50 individuals) were sighted during three or more primary sampling sessions (years), and 32% (21 individuals) were sighted consistently over the six-year period, including eight females and nine males (Fig 3). Over the 30 secondary sampling sessions (months), 44 individuals (66%) were captured on five or more occasions, 28 of which were captured on 10 or more occasions (Fig 4). Males tended to be sighted more often than females, with both sexes being sighted a maximum of 26 times out of 30 sampling sessions. Individuals of unknown sex tended to be sighted less frequently, five of them being observed only once.

### Assumption tests

Closure tests validated that the data collected within each five-month primary sampling session were consistent with a closed population, with no significant gain or loss of individuals detected (*p* > 0.01; Table 2). Similarly, the trap-dependence tests did not revealed any evidence of a behavioural response to capture (*p* > 0.01; Table 2), confirming that individuals had the same probability of being first captured and then subsequently recaptured (*p* = *c*).

**Table 2:** Results of the assumption tests on population closure and trap-dependence.

	Closure test		Trap-dependence test	
	*z*	*p*	*z*	*p*
2009	−0.68173	0.24802	3.0882	0.2135
2010	−0.71619	0.23694	0.1563	0.4528
2011	1.63010	0.94846	1.4952	0.4735
2012	−1.59317	0.05556	0.1579	0.9241
2013	1.97249	0.97572	5.5844	0.0612
2014	−2.19615	0.01404	1.4022	0.4905

### Robust Design models—All individuals combined

When combining all individuals (without distinction of sex), the most parsimonious and best-fitting model, based on the lowest AICc value, was [*ф*(.), *γ”*(.) = *γ’*(.), *p*(*t*,*T*), *N*(.)], referring to constant apparent survival, random and constant temporary emigration, time-varying capture probability (within and between primary sessions) and a constant population estimate (Table 3). This model provided an abundance estimate of 44.87 (SE = 0.548) distinct individuals, an annual apparent survival rate of 0.928 (SE = 0.019) and a temporary emigration rate of 0.098 (SE = 0.026). The second best-fitting model also supported the data (ΔAICc < 2; Table 3), but followed a Markovian temporary emigration pattern [*γ”*(.), *γ*’(.)]. This second model yielded similar estimates for survival and abundance parameters as the first model, but predicted a Markovian movement pattern of individuals in and out the survey area: the probability of being out of the survey area was estimated to be higher for individuals that were out in the previous primary sampling session (*γ’* = 0.199, SE = 0.139) than for those that were previously in (*γ”* = 0.095, SE = 0.026).

**Table 3:** Results of the top-ranked Robust Design models, based on the AICc scores, for all distinct individuals identified on the west coast of Reunion during June–October of 2009–2014.

Models	AICc	ΔAICc	Nb of Parameters	Deviance	Survival (ф)	Temporary emigration(*γ*”, *γ*’)	Abundance of distinctive individuals (*N*_*d*_)
(1) ф(.),*p*(*t*,*T*), *N*(.), *γ*’ = *γ*”(.) random	518.7	0	33	448.9	0.928 (±0.018, 0.883–0.957)	*γ*” = *γ*’ = 0.098 (±0.026, 0.058–0.163)	44.87 (±0.548, 44.28–46.70)
(2) ф(.),*p*(*t*,*T*), *N*(.), *γ*’(.), *γ*”(.) Markovian	520.2	1.5	34	438.7	0.930 (±0.018, 0.884–0.959)	*γ*” = 0.095 (±0.026, 0.055–0.161) *γ*’ = 0.199 (±0.139, 0.043–0.579)	44.87 (±0.548, 44.28–46.70)

AICc: Akaike Information Criterion, corrected for a small sample size;

ΔAICc: AICc difference from the first-ranked model;

Ф: apparent survival;

*γ*’,*γ*”: temporary emigration parameters;

*N*_*d*_: abundance of distinct individuals.

The probability of capture, which in the Robust Design approach refers to the probability of an individual to be both present in the survey area and captured (conditional of presence in the survey area), was highly variable between primary sampling sessions, ranging from 0.09 to 0.77 in both models. Models incorporating survey effort (number of survey trips and number of kilometers surveyed per primary sampling session) or marking level as a covariate to explain the variation in capture probability, showed a lower fit to the data (ΔAICc = 58.8, 54.8 and 81.2, respectively).

### Robust Design models—With gender groups

The Robust Design models run with females, males and individuals of unknown sex as separate groups showed that the best-fitting model was [*ф*(.), *γ*’_F_ = *γ*”_F_, *γ*’_M_ = *γ*”_M_, *p*(t,T), *N*(sex)] (Table 4). This model accounted for constant survival (over time and between sexes) and time-varying capture probability (within and between primary sessions). The first-ranked model described a random movement pattern for all groups, with the probability of temporary emigration of males being close to zero (*γ*”_M_ = *γ*’_M_ = 0.009, SE = 0.031). Two models ranked within less than two ΔAICc units of the first one, and thus were also considered to support the data. These models both predicted no temporary emigration for males (*γ*”_M_ and *γ*’_M_ fixed to zero) and random emigration for individuals of unknown sex, but differed in the movement pattern of females: the second-ranked model considered that the movement pattern of females was random, while the third-ranked model considered a temporally structured (Markovian: *γ*”_F_ ≠ *γ*’_F_) movement pattern of females. Thus, the three retained models showed null or quasi-null temporary emigration for males between primary sampling sessions, but differed in their temporary movement pattern for females. The first two models estimated that females followed a random movement pattern and had a probability (*γ*”_F_) of 0.10 (SE = 0.04) of being outside the survey area between each primary sampling session, regardless of whether they were previously present or absent (*γ*”_F_ = *γ*’_F_), and thus a probability of returning of 0.89 (return rate: 1 − *γ*’_F_). The third model predicted a Markovian temporary movement pattern for females. This model estimated a similar probability for females to emigrate from the survey area (*γ*”_F_ = 0.11, SE = 0.04) than previous models, but considered that the probability of staying out of the survey area over consecutive sampling sessions was null (*γ*’_F_ = 0), meaning that individuals that left the survey area in a given primary session re-entered the survey area in the next sampling session (return rate: 1 − *γ’*_*F*_ = 1). All three models predicted random and relatively higher estimates of temporary emigration for individuals of unknown sex (Table 4).

**Table 4:** Results of the Robust Design models, within two ΔAICc units from the first-ranked model, for distinct individuals identified on the west coast of Reunion, in June–October of 2009–2014, with females (F), males (M) and individuals of unknown sex (U) taken as distinct groups.

Models	AICc	ΔAICc	Nb Param.	Deviance	Survival (ф)	Temporary emigration (*γ*”, *γ*’)	Abundance of distinct individuals (*N*_*d*_) of males, females and unknown sex
**(1)** ф(.),*p*(*t*,*T*), *N*(sex)	1038.6	0	37	959.9	0.927 (±0.018, 0.882–0.956)		
Male: (*γ*’_M_ = *γ*”_M_)—Random						*γ*”_M_ = *γ*’_M_ = 0.009 (±0.031,0.001–0.895)	*N*_*d* M_ = 17.05 (±0.001, 17.00–17.01)
Female: (*γ*’_F_ = *γ*”_F_)—Random						*γ*”_F_ = *γ*’_F_ = 0.101 (±0.041, 0.044–0.218)	*N*_*d* F_ = 16.00 (±0.007, 16.00–15.01)
Unknown: (*γ*’_U_ = *γ*”_U_)—Random						*γ*”_U_ = *γ*’_U_ = 0.205(±0.061, 0.110–0.350)	*N*_*d* U_ = 11.00 (±0.003, 11.00–11.00)
**(2)** ф(.),*p*(*t*,*T*), *N*(sex)	1038.7	0.09	37	960.0	0.927 (±0.018, 0.882–0.955)		
Male (*γ*’_M_ = *γ*”_M_ = 0)—Null						*γ*”_M_ = 0 (fixed)	*N*_*d* M_ = 17.00 (±0.003, 17.00–17.01)
Female: (*γ*’_F_ = *γ*”_F_)—Random						*γ*”_F_ = *γ*’_F_ = 0.100 (±0.041, 0.044–0.214)	*N*_*d* F_ = 16.00 (±0.002, 16.00–16.01)
Unknown: (*γ*’_U_ = *γ*”_U_)—Random						*γ*”_U_ = γ’_U_ = 0.204 (±0.061, 0.109–0.345)	*N*_*d* U_ = 11.00 (±0.000, 11.00–11.00)
**(3)** ф(.),*p*(*t*,*T*), *N*(sex)	1040.0	1.31	38	958.9	0.926 (±0.018, 0.882–0.955)	*γ*”_M_ = 0 (fixed)	
Male: (*γ*’_M_ = *γ*”_M_ = 0)—Null						*γ*”_F_ = 0.109 (±0.043, 0.049–0.227)	*N*_*d* M_ = 17.00 (±0.001, 17.00–17.00)
Female: (*γ*’_F_ ≠ *γ*”_F_)—Markovian						*γ*’_F_ = 0 (fixed)	*N*_*d* F_ = 16.00 (±0.000, 16.00–16.01)
Unknown: (*γ*’_U_ = *γ*”_U_)—Random						*γ*”_U_ = γ’_U_ = 0.204(±0.061, 0.109–0.349)	*N*_*d* U_ = 11.00 (±0.001, 11.00–11.00)

AICc: Akaike Information Criterion, corrected for a small sample size;

ΔAICc: AICc difference from the first-ranked model;

Ф: apparent survival;

*γ*’,*γ*”: temporary emigration parameters;

*N*_*d*_: abundance of distinct individuals.

#### Proportion of distinct individuals and overall abundance estimates

The estimated proportion of distinct individuals in the population (*θ*) was 0.70 (SE = 0.03). This value of *θ* was used to compute the total number of individuals, *N* (including distinct and unmarked individuals), in the study area. Based on the estimated number of distinct individuals (*N*_*d*_ = 44.87) produced by the Robust Design approach, the total number of individuals (*N*) present along the west coast during the primary sampling sessions was 64.10 (SE = 5.8, 95%CI = 53.8–76.4). As the first ranked model described a random movement pattern of individuals, the extrapolated abundance estimate for the overall population of Reunion was *N°* = 71.1 individuals (SE = 6.05, 95%CI = 60.2–84.0), based on a proportion of 0.098 temporary migrants.

## Discussion

Because the entire range of the species around the island could not be systematically sampled, the Robust Design approach was used to produce the most accurate abundance and survival estimates to date for the Indo-Pacific bottlenose population of Reunion, based on a multilevel sampling of the west coast. This survey design provides an appropriate sampling scheme for deriving population parameters of this species around a small oceanic island, allowing the restriction of survey effort both spatially (i.e. west coast only) and temporally (i.e. five months each year), while accounting for individual movements around the island. The probability of capture was highly variable, both within and across primary sessions, but independent of survey effort (models using survey effort as a covariate to explain the variation in capture probability did not support the data), which suggests that sufficient effort was deployed and that the capture probability was mainly conditioned by presence in the survey area.

### Abundance estimates

This study presents the first abundance estimate of the Indo-Pacific bottlenose dolphin population for Reunion Island. Models presenting constant abundance over the years best supported the data, suggesting that the population was stable over the six years surveyed (2009–2014). The estimated number of individuals (distinct and unmarked) present along the west coast during primary sampling periods (June–October) was *N* = 64.1 (SE = 5.8, 95%CI = 53.8–76.4). When accounting for the proportion of temporary emigrants (0.098), the abundance of the entire population of Reunion was estimated to be *N°* = 71.1 (SE = 6.05, 95%CI = 60.2–84.0), based on a random movement pattern of individuals in and out the survey area [5]. However, because the temporally structured (Markovian) movement pattern model could not be entirely ruled out (Δ AICc < 2), the extrapolation of the entire population size (*N°*) might be slightly biased. The bias is believed to be small because both models (random and Markovian) produced similar estimates of temporary emigration (*γ*” = 0.098 and *γ*” = 0.095, respectively), indicating that almost 90% of the population (1 − *γ*” = 0.9) remained in the study area between consecutive primary sampling sessions. The Markovian model differed in predicting a higher probability of remaining outside the survey area (*γ*’ = 0.199) compared to the random model (*γ*’ = *γ*” = 0.098).

Despite the uncertainty in relation to movement patterns, it was deemed appropriate to extrapolate the size of the overall population (*N°*) to provide the most plausible abundance estimate for the local population of Reunion. In fact, for conservation purposes, it is important to provide an estimate for the entire island population (*N°*), rather than to report only on the number of individuals present in the survey area during June–October (*N*). This approach is relevant for remote island–associated populations of *T*. *aduncus* (and other inshore species), whose spatial range is expected to be confined to an insular habitat. In continental areas, where individual spatial range might be more difficult to determine, extrapolation of the overall population size associated with the survey area is generally not undertaken [6, 7, 8, 9, 10, 11, 12]. A similar approach was, however, used to estimate the abundance of the western grey whale population in the North Pacific [49].

The population size of *T*. *aduncus* in Reunion was found to be small (71 individuals, 95%CI: 60–84). This is consistent with abundance estimates produced for other oceanic islands of similar size, such as Mayotte and Mauritius [17, 18], although direct comparisons are difficult due to the different methods used (models and size of the survey area). For Reunion, the abundance estimate of the overall population refers to a spatial range of around 786 km^2^ (waters < 100 m deep). Around Mayotte, the survey effort covered the entire species range (848 km^2^) and closed-population models produced an estimate of 82 individuals (95%CI: 24–169) for the entire island [17]. For Mauritius, open-population models estimated that 59 individuals (95%CI: 54–63) were present in a survey area of around 100 km^2^ off the southwest coast (approximately 20% of the coastline) [18]. Although some individuals were shown to travel around the island, the proportion of individuals outside the survey area during sampling (temporary emigration) was not assessed and the overall population size for Mauritius could not be extrapolated [18]. In continental waters, very small populations of *T*. *aduncus* (< 100 individuals) have also been reported in restricted environments such as estuaries and enclosed bays [9, 11, 13, 15]. In East Australia, populations of 34 (95%CI: 19–49) and 71 (95%CI: 62–81) individuals were estimated to inhabit the estuaries of the Clarence (19 km^2^) and Richmond (89 km^2^) rivers, respectively [15]. Similarly, abundance estimates ranging from 61 to 160 distinct individuals were obtained for the enclosed bays of Port Stephens (140 km^2^) and Jervis Bay (102 km^2^), in East Australia [13]. Off Zanzibar (Tanzania), yearly estimates ranged from 136 to 179 individuals for a 26 km^2^ survey area [14]. These studies were based on closed-population models and temporary emigration could thus not be quantified, although some individuals were considered to be transient in the survey area [13,15]. In more recent studies, the Robust Design approach has been used in Australia to produce abundance estimates of Indo-Pacific bottlenose dolphins and report on temporary emigration from survey areas in a variety of locations. In West Australia, abundance estimates in a 120 km^2^ survey area off Bunbury ranged from 63 individuals (95%CI: 59–73) in winter to up to 139 individuals (95%CI: 134–148) in summer, with some high levels of variation observed in temporary emigration, reflecting seasonal movements in and out the area, likely driven by breeding and prey availability [9, 12]. In Shark Bay, estimates varied from 115 (95%CI: 105–126) to 208 (95%CI: 177–245) and relatively high temporary emigration rates were obtained (*γ”* = *γ*’ = 0.33–0.66), indicating that the overall population associated with the survey area (226 km^2^) was larger [8]. In Northwest Australia, abundance estimates of fewer than 60 individuals and up to 157 (95%CI: 133–186) were obtained for two bays of around 130 km^2^ (Cygnet Bay and Beagle Bay, Kimberley), with no temporary emigration detected [11]. Therefore, the abundance estimate of Indo-Pacific bottlenose dolphins for Reunion Island (786 km^2^), as well as Mayotte [17], is within the range of those reported for much smaller sites (< 200 km^2^), suggesting that the species occurs in lower densities around these remote islands. The relatively low abundance estimated for small oceanic islands might reflect their geographic isolation and possibly the restricted carrying capacity of the habitat—especially in young islands like Reunion, where the steep underwater relief confines shallow waters to a narrow band along the coast [26].

### Survival rate

The best-fitting model indicated that survival probability was constant over time, with an annual apparent survival of 0.928 (±0.019 SE, 95% CI: 0.883–0.957). This estimate falls within the range of annual survival rates available for the species in other areas. For instance, a similar rate was obtained in Mayotte (0.93 ± 0.059 SE), while a slightly higher annual rate (around 0.95) was estimated for populations in Shark Bay, Australia, and off Bangladesh [8, 17, 23]. Survival rates of up to 0.99 have been reported in some areas, such as off Bunbury, Southwest Australia, and Mauritius [12, 18], but these referred to seasonal or monthly survival rates, and are thus not directly comparable.

The survival rate of the present study is believed to represent the true annual survival rate of Indo-Pacific bottlenose dolphins at Reunion. Although apparent survival may result from both mortality and permanent emigration, the latter is believed to be very limited for this species at Reunion. Being resident around the island and constrained to coastal habitat [26], trans-oceanic migration is likely to be very rare, if not completely absent. Furthermore, the survival rate reported in this study is believed to refer mostly to adult animals. In fact, with dorsal fin marks being acquired with age, immature individuals were generally insufficiently marked [mark level (<2)] to be included in the capture–recapture analysis. No difference in survival between males and females was detected, which is consist with the few studies that have estimated sex-specific survival rates for Indo-Pacific bottlenose dolphins [9, 12].

The annual survival rate was relatively high, as expected in long-living mammals, and consistent between years, suggesting that the local population is not facing drastic and/or increasing sources of adult mortality. Although the species might be relatively more exposed to natural predation at Reunion [52], no major source of direct human-induced mortality has been identified. Interactions with local fisheries is not considered to have a significant impact on the population, as gillnet fisheries—mostly responsible for by-catches of coastal dolphins in the region [32]–are not used at Reunion, and lethal interactions with other fisheries have never been reported. Conversely, human-related disturbances, such as habitat degradation and fragmentation, and unregulated dolphin-watching activity are believed to represent the most important threat for the species at Reunion [26, 37], but their impacts at the population level are unknown and might be very difficult to detect.

### Temporary emigration and sex-specific movement pattern

Estimates of temporary emigration produced under the Robust Design approach are closely related to the sampling design and the size of the study area relative to the overall population range, which is usually unknown. Comparisons with estimates from other studies are therefore difficult to interpret. In Australia, where the Robust Design method has been used on Indo-Pacific bottlenose dolphin populations, temporary emigration reported for smaller study areas (around 100–200 km^2^) is highly variable, with γ’ ranging from 0 to 0.9 [8, 9, 11, 12]. The temporary emigration of 0.098 obtained in this study was relatively low, indicating that the study design (survey area and sampling period) allowed for the sampling of a large proportion of the population during each primary sampling session. Only a small proportion (9.8%) of the population was not available for capture when sampling the west coast of Reunion for five months every year, versus the entire habitat range year-round. This low level of temporary emigration also suggests high usage of the survey area, with most individuals (90.2%) being present along the west coast during the five-month sampling sessions. The west coast might thus represent a more suitable habitat compared to the east coast, which is more exposed to prevailing winds, and where depth increases very rapidly near the shore.

The models that discriminated between the sexes demonstrated differences in temporary emigration between males and females, suggesting sex-specific movement patterns and residency. The low but consistent temporary emigration detected for females (*γ”*_F_ = 0.1) indicated that they were not systematically present in the study area during each primary sampling session (June–October). Thus, while a majority of females might have used the west coast on a regular basis, a small proportion (10%) resided outside for several (more than 5) months. This suggests that some females might be using core areas outside the survey site, coming back less frequently to the west coast. The high return rate (1 − 0.1 = 0.9) indicated that most females that were absent from the survey area in a given primary sampling session (June–October) re-entered the survey area in the next one, suggesting females that are transient to the west coast still use the area on a yearly basis (every eight months at a minimum, and 18 months maximum). These movement patterns are consistent with the enhanced local residency of female bottlenose dolphins, which tend to concentrate their activity in some core areas, while showing a certain level of home-range overlap [20, 53, 54].

On the contrary, males showed null or quasi-null temporary emigration (*γ”*_M_ = 0.009 for the first-ranked model and *γ”*_M_ = 0 for the second- and third-ranked models), indicating they were available for capture in the study area during every primary sampling session. Given that males were also observed along the east coast (S1 Appendix), the null temporary emigration does not imply that they stay within the survey area at all times; rather, it depicted an overall null displacement, with individuals repeatedly returning to the survey area. Therefore, the null temporary emigration obtained for males demonstrates that they consistently use the west coast, with most probably adopting short-term movement patterns around the island. This is consistent with other Indo-Pacific bottlenose dolphin studies, in which it has been suggested that males disperse over larger home ranges and show lower site fidelity compared to females [20, 53, 54, 55, 56]. In West Australia, temporary emigration has been shown to vary seasonally, and the suggestion is that males follow the distribution of reproductively active females during the breeding season (summer/autumn), but increase their home range during the non-breeding season to optimize food resources [20]. As sampling took place during austral winter (June–October) at Reunion, the pattern described in this paper might refer to the non-breeding season, when the movement behaviour of males may be less influenced by mating. Seasonal changes in movement pattern would be worthy of further investigation in future work.

### Conservation implications and perspectives

The present study shows that Indo-Pacific bottlenose dolphins at Reunion form a small resident population, of fewer than 100 individuals, with apparently stable adult survival. In itself, the small size of the population makes the species highly vulnerable and warrants the implementation of a precautionary approach to its conservation. Population viability analysis has indicated that populations of fewer than 100 animals face a very high risk of extinction, being more vulnerable to environmental changes and demographic stochasticity [57, 58, 59, 60]. Furthermore, the geographic isolation of island-associated populations might increase their vulnerability to disturbance and raise concern about the long-term viability of *T*. *aduncus* in remote oceanic islands, where the open ocean most likely represents a barrier to migration and gene flow. Although no trend in abundance and survival was detected over the study period, signs of population decline might take several years to be detected in the field [61, 62, 63]. Besides, although adult survival is generally considered as having the greatest influence on the viability of slow-growing populations [64], recent studies conducted on the species demonstrated that population dynamics can also be greatly affected by changes in the vital rate [34], which was not addressed in this study. Future research should aim at investigating the reproductive rate and calf survival at Reunion in order to model long-term population trends. Modelling the population viability under alternative management scenarios would be particularly useful, to identify which conservation measures are likely to be most effective in ensuring the longevity of the population. In the meantime, management actions should aim at minimizing any source of potential impact of human activity on the local population and habitat of Indo-Pacific bottlenose dolphins.

## Supporting information

S1 Appendix Around-island surveys conducted off Reunion in 2013. (PDF)

S2 Appendix Formulae used to compute the standard error and confidence interval of *N* (number of estimates of distinct and unmarked individuals). (PDF)

S1 Table Monthly survey effort deployed over the study period (2009–2014), from June to October, off the west coast of Reunion, and photo-identification effort applied to *T*. *aduncus*. (PDF)

S1 Dataset Individual capture histories of distinct Indo-Pacific bottlenose dolphins photo-identified during June–October of 2009–2014 along the west coast of Reunion, and associated information (sex and marking level). (PDF)
