# Supplementary material for: Identifying key demographic parameters of a small island–associated population of Indo-Pacific bottlenose dolphins (Reunion, Indian Ocean)
Source: PLoS One. 2017 Jun 22;12(6):e0179780. doi: 10.1371/journal.pone.0179780 (PMC5480988; doi:10.1371/journal.pone.0179780)
Supplement: S1 Appendix — (PDF) [file pone.0179780.s001.pdf]

S1 Appendix. Around-island surveys conducted off Reunion in 2013.

In 2013, three surveys were conducted around the island to verify that no other population of *T. aduncus* was present outside the Robust Design study area (i.e. on the east coast). Daily trips were conducted around the island in waters less than 100m deep, during three sampling periods: from the 28/02/2013 to the 26/03/2013; from the 22/06/2013 to the 08/07/2013 and from the 07/10/2013 to the 10/11/2013, from different port of Reunion (Saint Gilles, Saint-Leu, Saint Pierre, Sainte Rose, Saint-Denis), using the same field methodology described in the paper.

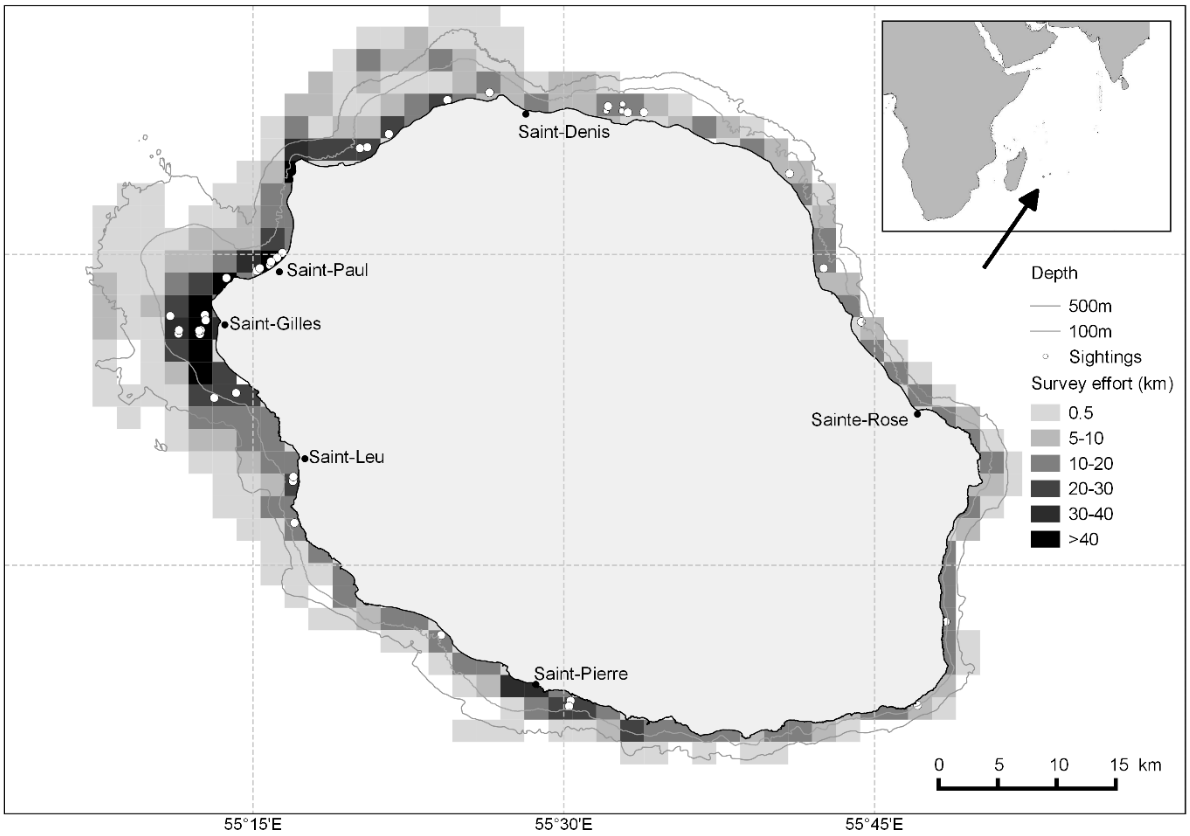

Figure A1. Map of Reunion Island representing Indo-Pacific bottlenose dolphin sightings and survey effort deployed during round-island surveys conducted in 2013.

Surveys were conducted over a period of 27 to 48 days, during which 8-9 daily boat trips were achieved (Table A1). A total of 39 *T. aduncus* sightings were reported around the island (Fig. A1), resulting in a cumulative number of 52 distinct individuals identified. All individuals identified during these surveys were also present in the Robust Design dataset (June–October of 2009-2014) collected along the west coast, meaning that round-island-surveys did not result in new identifications: all individuals sighted round the island and in particular on the east coast were also captured on the west coast at some point

during the Robust Design survey. Of the 21 distinct individuals sighted on the east coast (out of the Robust Design Study area), six were females, five were males and 10 were of unknown sex.

Table A1. Summary of the 3 surveys conducted around the island of Reunion in 2013 and details of *T.aduncus* sightings.

Survey period	Number of daily surveys	Number of sightings	Number of distinct individuals	Cumulative number of new identification
28 Feb-26 March	9	14	34	34
22 June-8 July	9	11	35	45
7 Oct.-10 Nov.	8	14	41	52

These data were used to produce abundance estimates using standard closed population models, taking each of the three round-island surveys as a distinct sampling occasion. Only high quality photographs showing well-marked individuals [marking level (2) or (3)] were considered. Closure tests, implemented in CloseTest ©, confirmed that the population sampled around the island in 2013 could be considered as closed to gain and loss of individuals (Otis test z=-1.0, p=0.158). Abundance estimates were produced using CAPTURE closed population models [42] implemented in Mark. The program CAPTURE select the model that best fits the data among a set of models, allowing capture probability to be constant (model Mo), time varying (model Mt) and/or to account for heterogeneity in capture probability between individuals, time varying or not (models Mth, Mh, respectively). The CAPTURE model selection procedure designated the model with constant probability of capture (Mo) as best-fitting the data and provided an abundance estimate of 53 (SE = 1.4, 95%CI: 53–59) distinct individuals present around the island during the survey period. The probability of capture between surveys was high (p=0.7). Based on a proportion of θ = 0.70 (SE = 0.03) distinct individuals in the population, the population size (N, including distinct and unmarked individuals) derived from the closed model was estimated to be 75.7 (SE = 7.03, 95%CI: 63.1–90.8). This estimate was consistent with the overall abundance estimate ($N°$) obtained from the Robust Design approach, although it provided a slightly larger confidence interval.
