# Supplementary material for: Identifying key demographic parameters of a small island–associated population of Indo-Pacific bottlenose dolphins (Reunion, Indian Ocean)
Source: PLoS One. 2017 Jun 22;12(6):e0179780. doi: 10.1371/journal.pone.0179780 (PMC5480988; doi:10.1371/journal.pone.0179780)
Supplement: S2 Appendix — (PDF) [file pone.0179780.s002.pdf]

S2 Appendix. Formulae used to compute the standard error and 95% confidence interval of *N* (estimate number of both distinct and unmarked individuals)

The standard error (SE) of the estimate number of all individuals (distinct and unmarked), N, was computed as follow:

$$SE(N) = \sqrt{N^2\left(\frac{SE(Nd)^2}{Nd^2} + \frac{1-\theta}{n\,\theta}\right)}$$

where Nd is the estimate number of distinct individuals (output from the model), θ is the proportion of distinct individuals in the population and n is the number of sightings used to estimate θ.

The 95% Confidence Interval was calculated with a lower limit of $N°_{low} = N° / C$ and an upper limit of $N°_{up} = N° \times C$, where:

$$C = \exp\left(1.96\sqrt{\ln\left(1 + \frac{SE(N)^2}{(N)^2}\right)}\right)$$
