# Supplementary material for: Identifying key demographic parameters of a small island–associated population of Indo-Pacific bottlenose dolphins (Reunion, Indian Ocean)
Source: PLoS One. 2017 Jun 22;12(6):e0179780. doi: 10.1371/journal.pone.0179780 (PMC5480988; doi:10.1371/journal.pone.0179780)
Supplement: S1 Table — (PDF) [file pone.0179780.s003.pdf]

**S1 Table. Monthly survey effort deployed over the study period (2009–2014), from June to October, off the west coast of Reunion and photo-identification effort applied to *T. aduncus*.**

| Year | Month | Number of daily surveys | Survey effort (in km) in <100m deep waters | Number of groups sighted | Number of distinct individuals | Number of new identifications | Cumulative number of new identifications |
|---|---|---|---|---|---|---|---|
| 2009 | 6 | 4 | 104 | 4 | 13 | 13 | 13 |
| | 7 | 28 | 555 | 7 | 20 | 13 | 26 |
| | 8 | 33 | 699 | 9 | 21 | 4 | 30 |
| | 9 | 26 | 682 | 7 | 20 | 3 | 33 |
| | 10 | 18 | 439 | 8 | 23 | 11 | 44 |
| 2010 | 6 | 5 | 117 | 5 | 17 | 2 | 46 |
| | 7 | 26 | 695 | 16 | 27 | 1 | 47 |
| | 8 | 43 | 1 008 | 6 | 11 | 1 | 48 |
| | 9 | 36 | 894 | 8 | 24 | 2 | 50 |
| | 10 | 31 | 812 | 12 | 22 | 2 | 52 |
| 2011 | 6 | 22 | 497 | 19 | 24 | 3 | 55 |
| | 7 | 32 | 647 | 19 | 26 | 1 | 56 |
| | 8 | 43 | 1 068 | 18 | 26 | 0 | 56 |
| | 9 | 25 | 651 | 7 | 12 | 0 | 56 |
| | 10 | 25 | 660 | 15 | 33 | 0 | 56 |
| 2012 | 6 | 21 | 615 | 11 | 25 | 3 | 59 |
| | 7 | 37 | 1 006 | 15 | 32 | 1 | 60 |
| | 8 | 37 | 960 | 13 | 32 | 1 | 61 |
| | 9 | 28 | 688 | 6 | 17 | 0 | 61 |
| | 10 | 19 | 633 | 10 | 27 | 1 | 62 |
| 2013 | 6 | 16 | 391 | 8 | 16 | 0 | 62 |
| | 7 | 43 | 929 | 12 | 23 | 0 | 62 |
| | 8 | 38 | 1 137 | 9 | 16 | 1 | 63 |
| | 9 | 20 | 413 | 10 | 11 | 0 | 63 |
| | 10 | 12 | 344 | 6 | 23 | 0 | 63 |
| 2014 | 6 | 16 | 288 | 9 | 15 | 1 | 64 |
| | 7 | 36 | 709 | 13 | 34 | 2 | 66 |
| | 8 | 38 | 854 | 17 | 25 | 0 | 66 |
| | 9 | 18 | 394 | 3 | 4 | 0 | 66 |
| | 10 | 11 | 270 | 3 | 11 | 0 | 66 |
| **TOTAL** | | **1173** | **19 161** | **305** | | **66** | |
