# Supplementary material for: Identifying key demographic parameters of a small island–associated population of Indo-Pacific bottlenose dolphins (Reunion, Indian Ocean)
Source: PLoS One. 2017 Jun 22;12(6):e0179780. doi: 10.1371/journal.pone.0179780 (PMC5480988; doi:10.1371/journal.pone.0179780)
Supplement: S1 Dataset — (PDF) [file pone.0179780.s004.pdf]

S1 Dataset. Individual capture histories of distinct Indo-Pacific bottlenose dolphins photo-identified during June−October of 2009−2014 along the west coast of Reunion, and associated information (sex and marking level).

Individual Capture history	Sex	Marking level
111110001100100011110000000100	Female	3
101110101111101010011100111100	Male	3
100111101101111111111111101101	Female	3
000010001001001001010000001000	Male	3
000000111000100000000000001101	Unknown	3
000000001100001000000000101101	Unknown	3
111010000000000000000000000000	Female	3
011110110111101111111111111100	Male	2
010000101011111111011100111100	Female	3
101001100100111111000100000110	Male	2
010100101111101111111111110110	Female	3
000100010000000100000100000000	Female	3
100111100100111111111110001111	Female	3
111001101011111011110000101100	Unknown	3
000000000000000101000100001000	Female	3
000011101110011111000000000000	Male	3
100100000011101000010000101001	Female	2
000011100010111011010010001100	Unknown	3
111001111011101011011110100100	Male	3
010000001011111111010101101100	Female	3
011101110011101011001110000100	Male	3
010000000000001000000000000000	Female	3
001100000000000100000000000000	Unknown	3
000110001000000000000000000000	Female	2
011101111011101011010011000000	Male	3
001011100000000000000000000000	Unknown	3
000011101111000001101000010100	Male	3
011101101111111110111111111100	Male	3
000010000101011111010100111000	Female	2
000000000100001110100000011000	Unknown	3
011101101111101110011111111100	Male	2
101001111101111111100110100010	Female	3
111100000000000000000000000000	Male	3
011110100100101000000001100000	Female	3
110100110000100011110000101100	Female	2
000010000000100000000000001000	Unknown	3
011010110111001011000000101000	Unknown	3
000010001001001001000000000000	Male	3
111011101000000110000000100000	Female	2
000010001000000000000000000000	Male	3

0001100001000001010000000000000	Unknown	3
0000000010000000000000000000000	Unknown	3
1011000000000000000000000000000	Unknown	2
0110001000110010111100000101000	Unknown	3
0100000000000000000000000000000	Unknown	3
0011001011111010100111001111100	Male	3
0000000001000000000100100001001	Female	3
0000100000100001111010100000001	Male	3
0000100000000000000000000000000	Unknown	2
0000100010010010000000000000000	Male	2
0000011101111011111111111111100	Male	3
0000011001000010010000001001001	Female	3
0000000100111010110100000001000	Male	3
0000000000111100011100000000000	Female	3
0000000000100001100010000000000	Male	3
0000000000100001110010100000001	Unknown	3
0000000000010011100000000001100	Unknown	3
0000000000000000000000000001100	Unknown	3
0000000000000000000101000001001	Unknown	2
0000000000000000100110100011100	Unknown	2
0000000000000000010001000110001	Unknown	3
0000000000000000101010100111100	Unknown	2
0000000000000000010000011001000	Unknown	2
0000000000000000000100101000000	Unknown	3
0000000000000000000000000001000	Unknown	3
0000000000000000000000000010000	Unknown	2
